# Intestinal Stem Cell Markers in the Intestinal Metaplasia of Stomach and Barrett’s Esophagus

**DOI:** 10.1371/journal.pone.0127300

**Published:** 2015-05-21

**Authors:** Bo Gun Jang, Byung Lan Lee, Woo Ho Kim

**Affiliations:** 1 Department of Pathology, Jeju National University Hospital, Jeju, South Korea; 2 Department of Anatomy, Seoul National University College of Medicine, Seoul, South Korea; 3 Department of Pathology, Seoul National University College of Medicine, Seoul, South Korea; Queen Mary Hospital, HONG KONG

## Abstract

Gastric intestinal metaplasia (IM) is a highly prevalent preneoplastic lesion; however, the molecular mechanisms regulating its development remain unclear. We have previously shown that a population of cells expressing the intestinal stem cell (ISC) marker *LGR5* increases remarkably in IM. In this study, we further investigated the molecular characteristics of these *LGR5* ^+^ cells in IM by examining the expression profile of several ISC markers. Notably, we found that ISC markers—including *OLFM4* and *EPHB2*—are positively associated with the *CDX2* expression in non-tumorous gastric tissues. This finding was confirmed in stomach lesions with or without metaplasia, which demonstrated that OLFM4 and EPHB2 expression gradually increased with metaplastic progression. Moreover, RNA in situ hybridization revealed that *LGR5* ^+^ cells coexpress several ISC markers and remained confined to the base of metaplastic glands, reminiscent to that of normal intestinal crypts, whereas those in normal antral glands expressed none of these markers. Furthermore, a large number of ISC marker-expressing cells were diffusely distributed in gastric adenomas, suggesting that these markers may facilitate gastric tumorigenesis. In addition, Barrett’s esophagus (BE)—which is histologically similar to intestinal metaplasia—exhibited a similar distribution of ISC markers, indicating the presence of a stem cell population with intestinal differentiation potential. In conclusion, we identified that *LGR5* ^+^ cells in gastric IM and BE coexpress ISC markers, and exhibit the same expression profile as those found in normal intestinal crypts. Taken together, these results implicate an intestinal-like stem cell population in the pathogenesis of IM, and provide an important basis for understanding the development and maintenance of this disease.

## Introduction

Preneoplastic intestinal metaplasia (IM) is associated with an increased risk of gastric carcinoma and presents in approximately one-fourth of individuals worldwide.[1] IM often results from chronic atrophic gastritis following infection with *Helicobacter pylori*, which can then advance to gastric epithelial dysplasia or carcinoma.[2] A variety of genetic and epigenetic alterations have been implicated in the pathogenesis of human IM.[3] Furthermore, long-term IM induced by CDX2 expression has been shown to lead to gastric cancer in transgenic mice, indicating that IM itself plays a significant role in the genesis of gastric carcinoma.[4]

Because IM is a critical precursor in gastric carcinogenesis, the potential to reverse these lesions is of great interest.[5] Previous investigations have reported that eradication of *H*. *pylori* is sufficient to reverse IM, yet others have found that a significant proportion of patients still present with IM even after effective eradication.[5] IM is believed to be the ‘point of no return’ in the histological cascade from chronic gastritis to adenocarcinoma;[6] thus, efforts to understand the molecular mechanisms regulating the establishment and maintenance of IM are crucial to develop strategies to interrupt gastric carcinogenesis. For instance, CDX2 autoregulation is suggested to have a major impact on the stability of IM lesions.[7] While IM crypts in the human stomach are clonal and contain multipotent stem cells,[8] it remains poorly understood whether native gastric stem cells are the initial source of metaplasia or if they only serve to maintain established lesions.

The discovery of normal gastric mucosal stem cells coincided with identification of the Wnt target gene *LGR5* as a stem cell marker in the intestinal epithelium.[9] A lineage-tracing study later revealed that *LGR5* ^+^ cells are multipotent stem cells responsible for renewal of the gastric epithelium in the mice.[10] Our group previously demonstrated that a small number of *LGR5* ^+^ cells also reside at the bottom of human antral glands and increase dramatically in IM lesions.[11] These findings led us to speculate that *LGR5* may be a marker for intestinal stem cells (ISCs) involved in the maintenance of IM.

Barrett’s esophagus (BE) is a metaplastic conversion to intestinal columnar epithelium and is associated with an increased risk of adenocarcinoma, similar to that observed with gastric IM.[12] Notably, human BE lesions exhibit an upregulation of *LGR5* expression when compared to normal squamous epithelium, and is suggestive of the presence of a *LGR5* ^+^ stem cells in BE.[13]

Several molecular ISC markers have been identified in addition to *LGR5*, including *PROM1* [14], *BMI1* [15], *LRIG1* [16], and *ASCL2*, which was identified as a transcription factor to control intestinal stem cell fate.[17] In addition, *OLFM4* [17] and *EPHB2* [18] are also highly expressed in ISCs. In this study, we aimed to discover additional ISC markers involved in the genesis and maintenance of gastric IM and BE, and examine their colocalization with *LGR5* ^+^ cells by RNA in situ hybridization to further reveal the molecular characteristics of *LGR5* ^+^ cells in IM with regards to the intestinal-like stem cell phenotype.

## Materials and Methods

### Subjects

Formalin-fixed and paraffin-embedded (FFPE) gastric samples with or without intestinal metaplasia (IM) were collected from five patients who underwent endoscopic submucosal dissection at Seoul National University Hospital (SNUH) from 2008 to 2010. IM lesions were categorized into gastric-and-intestinal mixed (GI) and solely intestinal (I) subtypes (also known as incomplete and complete types, respectively).[19] Samples of Barrett’s esophagus were isolated from two patients with adenocarcinoma of gastroesophageal junction, and a normal small intestine specimen was obtained from a patient with colon cancer. Fresh-frozen non-tumorous gastric tissues were available from 28 gastric cancer patients who had undergone surgical gastrectomy from 2001 to 2005 at SNUH.

### Ethical statement

All human specimens were obtained through curative surgical resection. This retrospective study was performed using stored samples after pathologic diagnosis. Samples were anonymized prior to the study, thus written consent was not required. The study design was approved by the Institutional Review Board at Seoul National University Hospital under the condition of anonymization (reference: H-1209-037-424).

### RNA in situ hybridization

In situ hybridization for *LGR5*, *ASCL2*, *OLFM4*, and *EPHB2* was carried out with the RNAscope FFPE assay kit (Advanced Cell Diagnostics, Inc., Hayward, CA, USA) as described previously.[11] Positive stain was defined as the presence of brown punctate dots in the nucleus and/or cytoplasm. The ubiquitin C and bacterial *DapB* genes served as positive and negative controls, respectively.

### RNA extraction and quantitative real-time PCR

Total RNA was extracted from paraffin-embedded tissue sections with an RNeasy FFPE Kit (Qiagen, Valencia, CA, USA) as previously described.[20] Reverse-transcribed cDNA was prepared from 1–2μg of total RNA with random hexamer primers and the GoScript reverse transcription system (Promega, Madison, WI, USA). Quantitative real-time PCR (qRT-PCR) reactions were performed using Premix EX Taq (Takara Bio, Shiga, Japan) according to the manufacturer’s recommendations, and the data analyzed using Sequence Detection System software (Version 1.4, Applied Biosystems). The following TaqMan gene expression assays were used: Hs00173664_m1 (*LGR5*), Hs00362096_m1 (*EPHB2*), Hs00270888_s1 (*ASCL2*), Hs00197437_m1 (*OLFM4*), Hs01009250_m1 (*PROM1*), Hs00394267_m1 (*LRIG1*), Hs00995536_m1 (*BMI1*), Hs00178027_m1 (*DCLK1*), Hs010780810_m1 (*CDX2*), Hs00212584_m1 (*CLDN18*), and Hs0275899_g1 (*GAPDH*). *GAPDH* served as the endogenous control.

### Transfection of CDX2

CDX2 cDNA (pCMV6-CDX2) was purchased from OriGene (Rockville, MD, USA). Gastric cancer cells were seeded at 1 × 10^6^ cells/well in 6-well plate and transfected with 2.5 μg of cDNA or empty control vector using Lipofectamine 2000 transfection reagent (Invitrogen, Carlsbad, CA, USA) according to the manufacturer’s instructions. Cells were subjected to qRT-PCR analysis approximately 24 h after transfection.

### Statistical analysis

Statistical analyses were performed in Prism 5 (GraphPad Software, Inc., San Diego, CA, USA). Correlations between the expressions of intestinal stem cell markers and *CDX2* was assessed by linear regression analysis. Mean differences between the groups of FFPE gastric specimens were assessed by one-way ANOVA. Between-group comparisons after transfection of *CDX2* in gastric cancer cell lines were performed using Student *t*-tests. The results were considered significant when *p* < 0.05.

## Results

### 1. ISC markers correlate with CDX2 levels in the gastric mucosa

**Fig 1:**
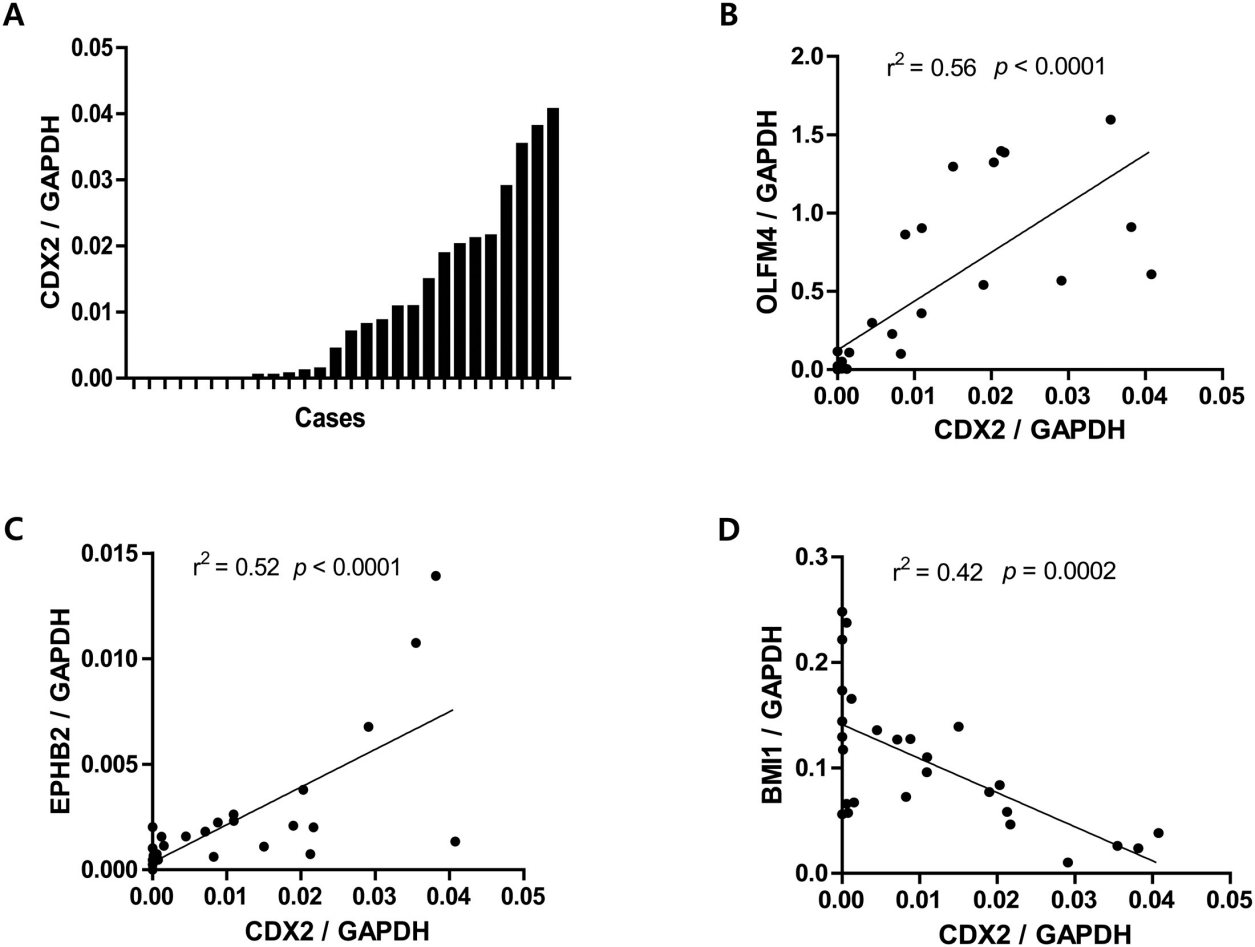
Correlation of *CDX2* and intestinal stem cell (ISC) marker expression in gastric tissues. *CDX2* and ISC marker expression in 28 fresh frozen-non-tumorous gastric tissues by quantitative real-time PCR (qRT-PCR) analysis. (A) Gastric mucosae show a wide range of *CDX2* expression levels, representing the various degrees of intestinal metaplasia. *OLFM4* (*p* < 0.0001, r^2^ = 0.56) (B) and *EPHB2* (*p* < 0.0001, r^2^ = 0.52) (C) expression increases significantly along with *CDX2* levels, whereas *BMI1* (*p* = 0.0002, r^2^ = 0.42) (D) decreases with *CDX2* expression.

We previously reported on the relative increase of *LGR5* ^+^ cells in IM lesions of human stomach.[11] This prompted us to hypothesize that these *LGR5* ^+^ cells may act as self-renewing stem cells to assist in the maintenance and propagation of metaplastic epithelium in the gastric mucosa. Thus, we aimed to identify additional ISC markers that correlated with *CDX2* expression in IM. For this, we measured the expression levels of *CDX2* and eight ISC markers—*LGR5*, *ASCL2*, *OLFM4*, *EPHB2*, *PROM1*, *DCLK1*, *LRIG1*, and *BMI1*—in normal gastric tissue. The examined tissues showed a wide range of *CDX2* levels, representing the various degrees of IM, since *CDX2* expression is positively correlated with IM progression (Fig 1A). Three ISC markers were found to correlate with *CDX2* expression: *OLFM4*, *EPHB2*, and *BMI1*. In particular, *OLFM4* (*p* < 0.0001, r^2^ = 0.56) (Fig 1B) and *EPHB2* (*p* < 0.0001, r^2^ = 0.52) (Fig 1C) displayed a strong positive correlation with *CDX2*, whereas *BMI1* was inversely correlated (*p* = 0.0002, r^2^ = 0.42) (Fig 1D). No significant association with *CDX2* expression was identified with the other five ISC markers (S1 Fig).

### 2. ISC marker expression correlates with IM progression

**Fig 2:**
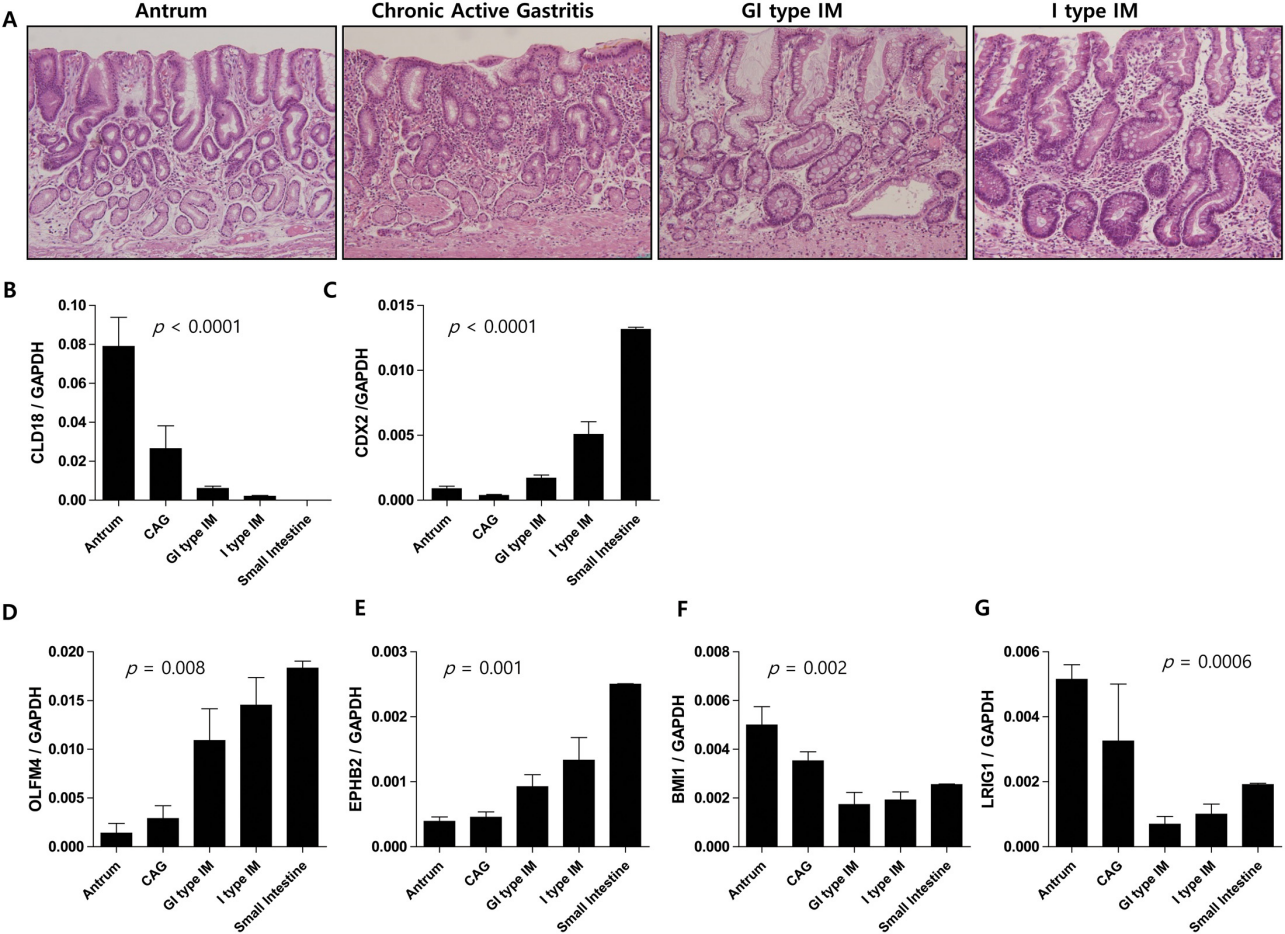
Altered expression of ISC markers coincides with gastric intestinalization. ISC expression in histologically distinct gastric lesions (n = 20), including normal antrum without intestinal metaplasia (IM) (n = 4), chronic active gastritis without IM (n = 3), gastro-intestinal mixed (GI) type IM (n = 6), solely intestinal (I) type IM (n = 5), and small intestine (n = 2). Hematoxylin and eosin staining of each gastric lesion (Magnification ×100, A). Claudin 18 expression gradually decreases (*p* < 0.0001, B), while CDX2 (*p* < 0.0001, C) increases with intestinal metaplastic progression. Among ISC markers, *OLFM4* (*p* = 0.008, D) and *EPHB2* (*p* = 0.001, E) expression gradually increases with metaplasia while *BMI1* (*p* = 0.002) (F) and *LRIG1* (*p* = 0.0006, G) expression is reduced.

To confirm the positive association of *OLFM4* and *EPHB2* with IM, we selected four histologically-distinct gastric tissue types; normal antral mucosa without IM (n = 4), chronic active gastritis without IM (n = 3), gastric and intestinal mixed type (GI type) IM (n = 6), and solely intestinal type (I type) IM (n = 5) (Fig 2A). Claudin-18 is the most highly expressed tight junction protein in the stomach. As expected, our analyses revealed that claudin-18 expression diminished with the increasing degrees of IM (*p* < 0.0001) (Fig 2B), whereas *CDX2* expression increased (*p* < 0.0001) (Fig 2C). We also found that *OLFM4* and *EPHB2* levels increased consistently with each subsequent lesion, confirming that these markers are closely related to IM progression (*p* = 0.008 and 0.001, respectively; Fig 2D and 2E). In contrast, *BMI1* and *LRIG1* expression showed a tendency to decrease with IM progression (*p* = 0.002 and 0.0006, respectively; Fig 2F and 2G).

### 3. *LGR5* ^+^ cells in intestinal metaplasia colocalize with other ISC markers

**Fig 3:**
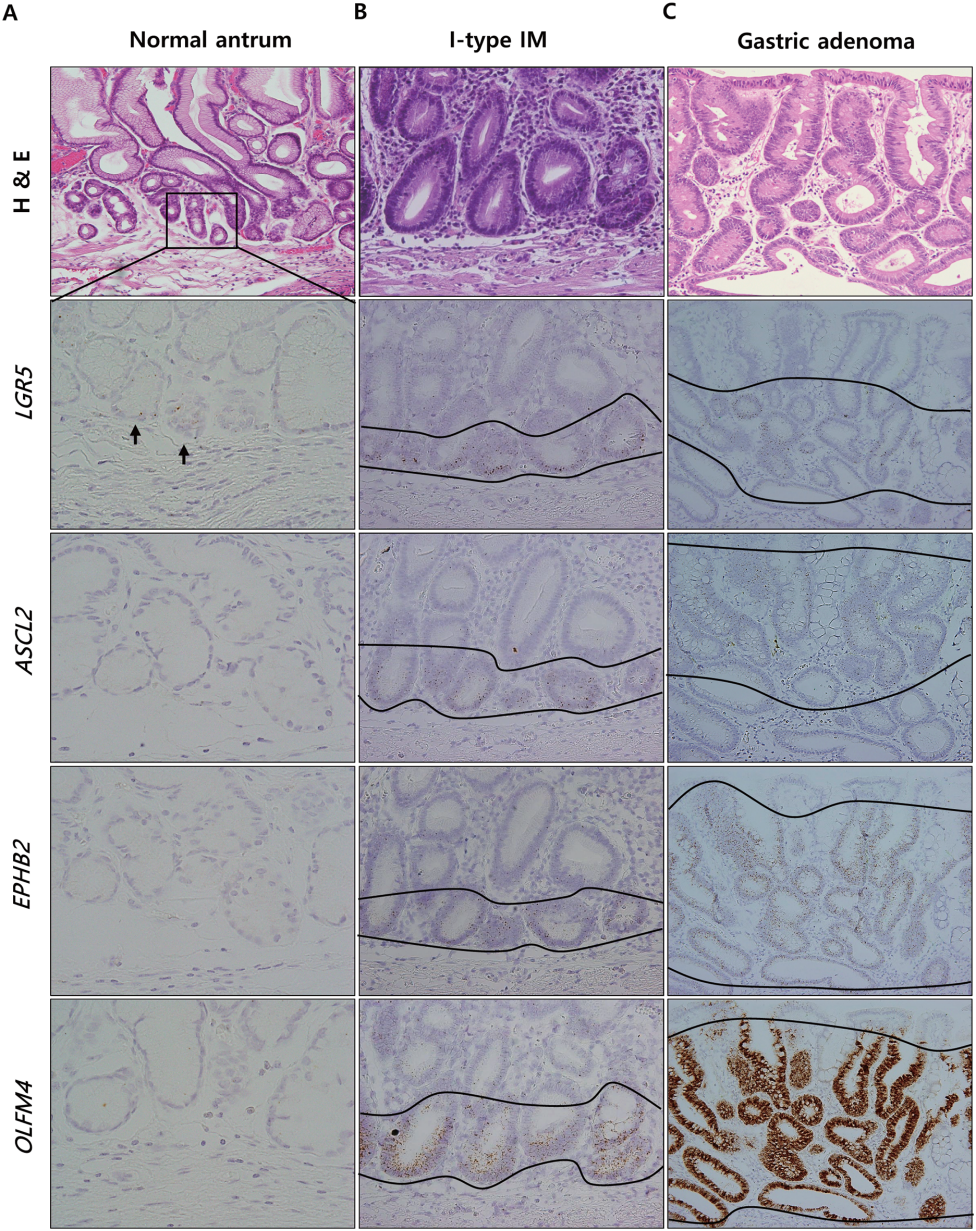
ISC marker expression in normal antrum, IM, and gastric adenoma. Representative H&E staining and in situ hybridization in IM and gastric adenoma with low grade dysplasia. *LGR5* ^+^ cells in normal antrum are devoid of ISC marker expression (A), whereas those in I-type IM located at the base of glands coexpress *ASCL2*, *EPHB2*, and *OLFM4* (B). Relative increase of *LGR5* ^+^ cell population with ISC marker expression (C) in gastric adenoma. Magnification: A (except H & E staining) ×100, B ×400; C ×200.

To determine if a direct relationship existed between *LGR5* and ISC markers in IM, we examined whether their coexpression by RNA in situ hybridization. The expression of *LGR5*, *ASCL2*, *EPHB2*, and *OLFM4* were first examined in a normal human small intestine section to validate this technique, and were found to localize specifically to cells within the stem cell niche of intestinal crypts as expected (S2 Fig). Since *LGR5* ^+^ cells in normal intestinal crypts also express ISC markers, we theorized that *LGR5* ^+^ cells in IM might also exhibit a similar expression pattern. Thus, we repeated the procedure on consecutive sections of endoscopic submucosal dissection specimens (n = 5), each of which contained multiple foci of GI- or I-type IM amongst non-tumorous tissue. A small number of *LGR5* ^+^ cells at the base of normal antral glands were devoid of ISC marker expression (Fig 3A), whereas those in I-type IM showed expressed all three markers (Fig 3B). Moreover, GI-type IM lesions displayed the same expression profile overall except for that ISC marker expression was located above the remaining gastric glands, rather than restricted to the basal areas (S3 Fig). This expression pattern was consistently observed in all samples. These findings indicate that *LGR5* ^+^ cells in IM differ from those in normal antrum in the expression of ISC markers that are usually restricted to cells within the intestinal crypts. Interestingly, IM derived from the fundic glands, where *LGR5* ^+^ cells are not normally present, produced the same results (S4 Fig). Thus, it seems likely that the population of *LGR5* ^+^ cells in IM does not result from the proliferation of preexisting *LGR5* ^+^ cells, but is rather an emergence of *LGR5* ^+^ cells with acquired differentiation potential, suggesting that an intestinal-like stem cell population is established in IM. In addition, gastric adenomas (n = 5) also expressed high levels of ISC markers throughout the lesions, rather than confined to the glandular crypts (Fig 3C). As shown in Fig 3, this accumulation of ISC marker-expressing cells over the metaplasia to dysplasia sequence is implicative of the participation of intestinal type stem cells in gastric tumorigenesis and additional studies to investigate the link between intestinal stem cell markers and gastric tumor development are certainly warranted.

### 4. ISC markers are expressed in Barrett’s esophagus lesions

**Fig 4:**
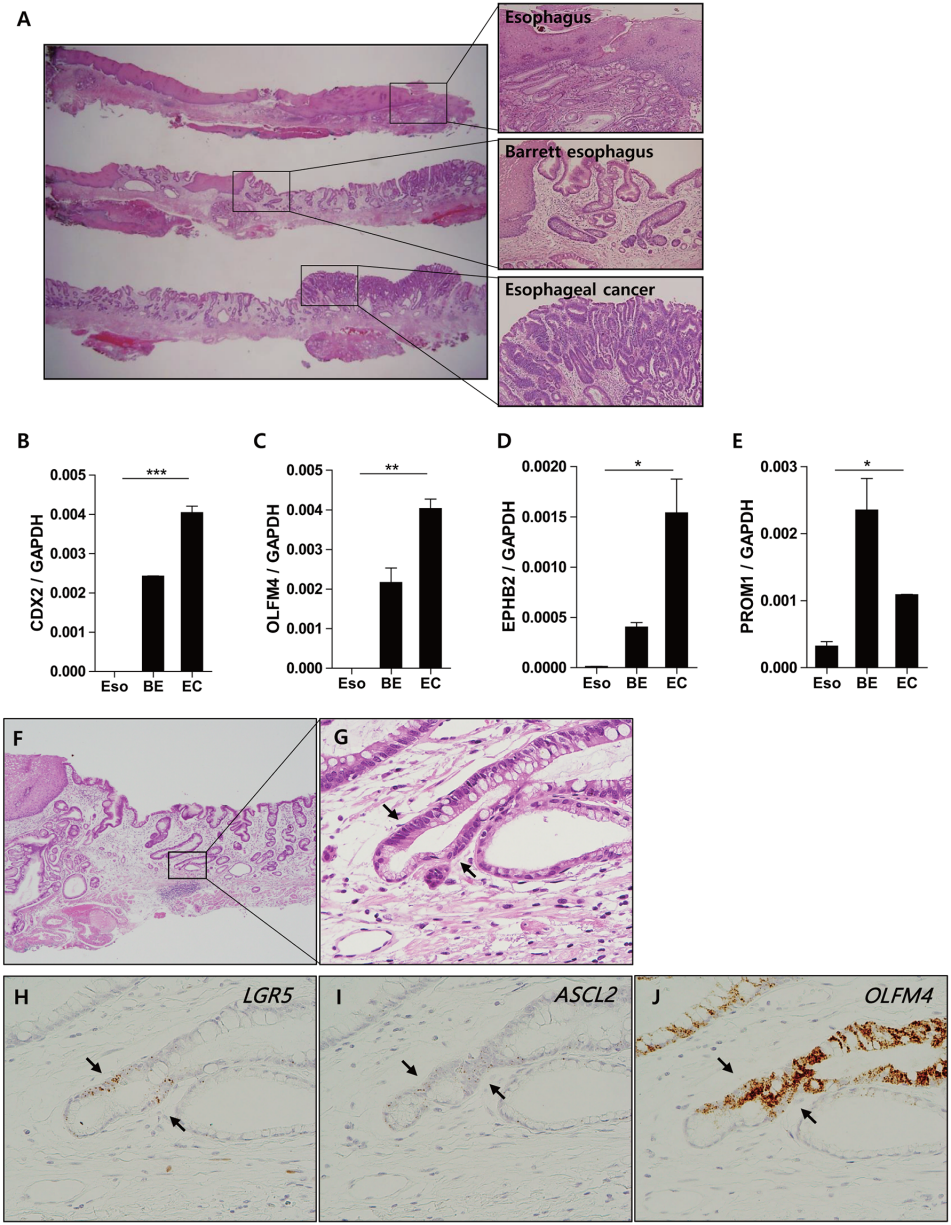
ISC marker expression in Barrett’s esophagus (BE). Both BE and adenocarcinoma sections from patients with adenocarcinoma of gastroesophageal junction (A) (n = 2) express higher levels of *CDX2* (***, *p* < 0.005) (B) and intestinal stem cell (ISC) markers including *OLFM4* (**, *p* < 0.01) (C), *EPHB2* (*, *p* < 0.05) (D), and *PROM1* (*, *p* < 0.05) (E) compared to normal esophageal mucosa. RNA in situ hybridization demonstrates that *LGR5* expression in basal metaplastic glands (G) colocalizes with *ASCL2* (H) and *OLFM4* (I). (A) and (F) show representative sections stained with hematoxylin and eosin. Arrowheads refer to intestinal-like stem cells that express all three markers. Magnification: A, ×1.25; F ×40; G-I ×400. Eso, esophagus; BE, Barrett’s esophagus; EC, esophageal cancer.

We next sought to determine whether ISC markers were also expressed in Barrett’s esophagus (BE). For this, two specimens of adenocarcinomas arising in the background of BE were assessed for the expression of *CDX2*, *OLFM4*, *EPHB2*, and *PROM1* by qRT-PCR analysis (Fig 4A). Significantly, both BE and adenocarcinomas expressed higher levels of all four ISC markers when compared to that of normal squamous epithelium (Fig 4B, 4C, 4D and 4E). While *LGR5* and *ASCL2* showed no significant changes from RT-PCR analysis, likely due to low copy number of transcripts, RNA in situ hybridization showed a clear cell population with *LGR5*, *ASCL2*, and *OLFM4* expression at the junction of the gastric and metaplastic glands, reminiscent of GI-type IM (Fig 4F, 4G, 4H, 4I and 4J).

## Discussion

The isthmus/neck region of the gastric unit was previously thought to encompass the stem cell niche in which IM occurs, and it has been suggested that metaplasic progression from a single gastric clonal unit occurs through clonal expansion and crypt fission.[8] because *LGR5* ^+^ cells are identified as multipotent intestinal-like stem cells in mice [10] and exist in the human gastric antrum [11], it is plausible to think that IM could develop from this cell population. However, there remains a possibility that another stem cell population could underlie this process. For example, *SOX2* ^+^ cells have also been identified as distinct gastric stem cells, and label cell population exclusive to those with *LGR5* expression.[21] Transdifferentiation can also give rise to metaplastic cells. Spasmolyitc polypeptide-expressing metaplasia—in which pyloric type glands appear in oxyntic mucosa—arise from mature chief cells, [22] rather than *LGR5*-expressing cells.[23] Indeed, transgenic *CDX1* or *CDX2* expression results in parietal cell-derived IM development in transgenic mice.[24] Therefore, it remains elusive whether IM is a consequence of intestinal stem cell reprogramming or the transdifferentiation of cells with acquired ISC-like properties.[12, 25]

Various efforts have sought to classify IM of stomach lesion types. Matuskura et al. suggested a classification system based on the presence of small intestine digestive enzymes; defined as complete and incomplete type IM,[26] whereas Jass and Filipe introduced three grades of IM on the basis of morphology and histochemical mucin staining. [27] More recently, a new classification has been proposed by Tatematu et al., in which IM can be divided into gastric-and-intestinal mixed (GI) and solely intestinal (I) types.[19] In GI-type IM, gastric and intestinal phenotypic markers appear at both the glandular and cellular levels, thus it has been suggested that IM might be caused by the gradual intestinalization of stem cells from the GI- to I-type.[28] Here, we observed gradual increase of the ISC markers *OLFM4* and *EPHB2* with further intestinalization of gastric mucosa. Along with the increasing *CDX2* levels that induce intestinal differentiation and phenotype, these expression patterns suggest a conversion of the overall stem cell population toward a more intestinal-like stem cell phenotype. Moreover, the unexpected inverse correlation of *BMI1* and *LRIG1* with IM needs further studies to confirm this result and to clarify its clinical implications.

*H*. *pylori* eradication has the potential to prevent gastric cancers,[29] and might attenuate the progression of precancerous gastric lesions, such as IM.[30, 31] However, once established, it appears that *H*. *pylori* eradication cannot completely prevent gastric cancer.[6] In fact, approximately 80% of subjects with IM showed no change or progression of IM after treatment with antibiotics.[31] In addition, a meta-analysis concluded that *H*. *pylori* eradication has no effect on gastric IM.[32] This irreversibility of IM could be partly explained by the maintenance of *CDX2* expression through an autoregulation loop that is independent of initial trigger, sustaining the intestinal phenotype.[33] We further revealed that *LGR5* ^+^ cells at the base of metaplastic glands consistently express other ISC markers, indicative of an intestinal-like stem cell phenotype. We believe that this stable stem cell population may provide an additional explanation for the long-lasting nature of IM. In addition, therapeutic strategies sufficient to specifically target the *LGR5* ^+^ cell population combined with *H*. *pylori* eradication could potentially undermine the stability of IM, thus accelerate the re-establishment of normal gastric mucosa.

Barrett’s esophagus (BE) is a precancerous lesion that shares several morphologic and molecular characteristics with gastric IM, mostly since it is a metaplastic conversion to intestinal columnar epithelium resulting from chronic inflammation. We believe that the present study characterizes another similarity between these two lesions in the presence of *LGR5* ^+^ cells with ISC marker expression. This finding is consistent with a previous report showing that *LGR5* expression was significantly elevated in BE, and that population is the likely cell-of-origin for this metaplasia.[13] More recently, *LGR5* ^+^ cells were identified in the middle of Barrett’s glands by in situ hybridization and are suggested to act as stem cells, as they exhibit both gastric and intestinal differentiation.[34] We also found *LGR5* ^+^ cells at the areas between gastric and metaplastic glands in BE, which correspond to the middle of Barrett’s glands. Additionally, the presence of ISC markers in the *LGR5* ^+^ cell population further supports their potential for intestinal differentiation. Thus, based on these results, it seems reasonable to suggest that *LGR5* ^+^ cells in BE likely function as stem cells that sustain the intestinal phenotype of BE, similar to that seen in IM.

CDX2 is a master transcription factor for the expression of intestinal differentiation markers, and is thought to underlie the development of BE. While normal gastric mucosa does not express *CDX2*, strong expression is detected in IM.[35, 36] Moreover, transgenic mice have demonstrated that *CDX2* expression alone is sufficient to induce IM [37, 38], suggesting that *CDX2* may also facilitate the development of stem cell population with an intestinal phenotype. Thus, we examined if *CDX2* is directly involved in the expression of the ISC markers: *LGR5*, *ASCL2*, *OLFM4*, and *EPHB2* (S5 Fig). However, transfection experiments revealed that only *EPHB2* was marginally affected by *CDX2* expression. Certainly, these data should be interpreted with caution since were obtained in GC cell lines with different biological properties from that of non-tumorous gastric epithelial or intestinal stem cells. Nevertheless, it seems likely that additional signaling factors along with *CDX2* are essential to induce ISC marker expression.

In summary, we determined that *LGR5* ^+^ cells in gastric IM and BE coexpress ISC markers, which is indicative of an intestinal-like stem cell population that replaces the preexisting gastric stem cells. This finding seems to provide an important clue for understanding the mechanism underlying the persistence of IM after *H*. *pylori* eradication. Furthermore, our findings suggest *LGR5* ^+^ cells are a promising target to reverse IM, and potentially prevent their progression into gastric cancers.

## Supporting Information

S1 Fig Correlation of intestinal stem cell (ISC) markers with *CDX2* levels in non-tumorous gastric tissues. No correlation is found between *CDX2* expression and some ISC markers such as *LGR5* (r^2^ = 0.01, *p* = 0.59), *ASCL2* (r^2^ = 0.01, *p* = 0.59), *PROM1* (r^2^ = 0.11, *p* = 0.08), *LRIG1* (r^2^ = 0.06, *p* = 0.21) and *DCLK1* (r^2^ = 0.09, *p* = 0.11).
(PPTX)

S2 Fig Visualization of intestinal stem cell markers by RNA in situ hybridization (ISH). RNA ISH performed on a formalin-fixed and paraffin-embedded specimen of small intestine. (A, B) A group of *LGR5* ^+^ stem cells are identified at the bottom of all crypts, intermingled with Paneth cells. Other intestinal stem cell markers such as *ASCL2* (C, D), *EPHB2* (E, F), and *OLFM4* (G, H) are also found to be confined to the crypt bases. Magnification: A, C, E, G ×100; B, D, F, H ×400.
(PPTX)

S3 Fig Expressions of intestinal stem cell markers in GI type IM. Remaining gastric glands are frequently found at the basal areas of GI type IM (A and B). RNA ISH shows that *LGR5* (C) and *EPHB2* (D) expressions are localized above the gastric glands. Interestingly, *OLFM4* (E) expression is observed in the gastric glands as well although its intensity is much weaker than that in the metaplastic glands. When those gastric glands disappear as IM develops (A and F), the distribution of all *LGR5* (G), *EPHB2* (H) and *OLFM4* (I) is strictly confined to the basal areas. Arrows indicate the remaining gastric glands. Magnification: A ×40; B, C, D, E, F, G, H, I ×200.
(PPTX)

S4 Fig Intestinal stem cell markers in intestinal metaplasia (IM) of the gastric corpus. (A and B) A small focus of IM in the middle of fundic glands, indicated by arrows, shows the same expression patterns of *LGR5* (C), *ASCL2* (D), and *OLFM4* (E) as the IM of antrum. Magnifications: A ×100; B, C, D, E ×200.
(PPTX)

S5 Fig Effect of *CDX2* on the expression of intestinal stem cell (ISC) markers in gastric cancer (GC) cell lines. Transfection of *CDX2* into four GC cell lines, MKN74 (A), MKN28 (B), SNU484 (C) and SNU668 (D) significantly increases the amount of mRNA of *CDX2* (**, *p* < 0.01; ***, *p* < 0.005). The *EPHB2* expression is only marginally enhanced by the expression of *CDX2* in three of four GC cell lines (***, *p* < 0.005). No difference is found in the levels of *LGR5*, *ASCL2*, and *OLFM4* upon *CDX2* overexpression (ns, not significant).
(PPTX)
