# Supplementary material for: Intestinal Stem Cell Markers in the Intestinal Metaplasia of Stomach and Barrett’s Esophagus
Source: PLoS One. 2015 May 21;10(5):e0127300. doi: 10.1371/journal.pone.0127300 (PMC4440782; doi:10.1371/journal.pone.0127300)
Supplement: S1 Fig — No correlation is found between CDX2 expression and some ISC markers such as LGR5 (r2 = 0.01, p = 0.59), ASCL2 (r2 = 0.01, p = 0.59), PROM1 (r2 = 0.11, p = 0.08), LRIG1 (r2 = 0.06, p = 0.21) and DCLK1 (r2 = 0.09, p = 0.11). (PPTX) [file pone.0127300.s001.pptx]

## Slide 1
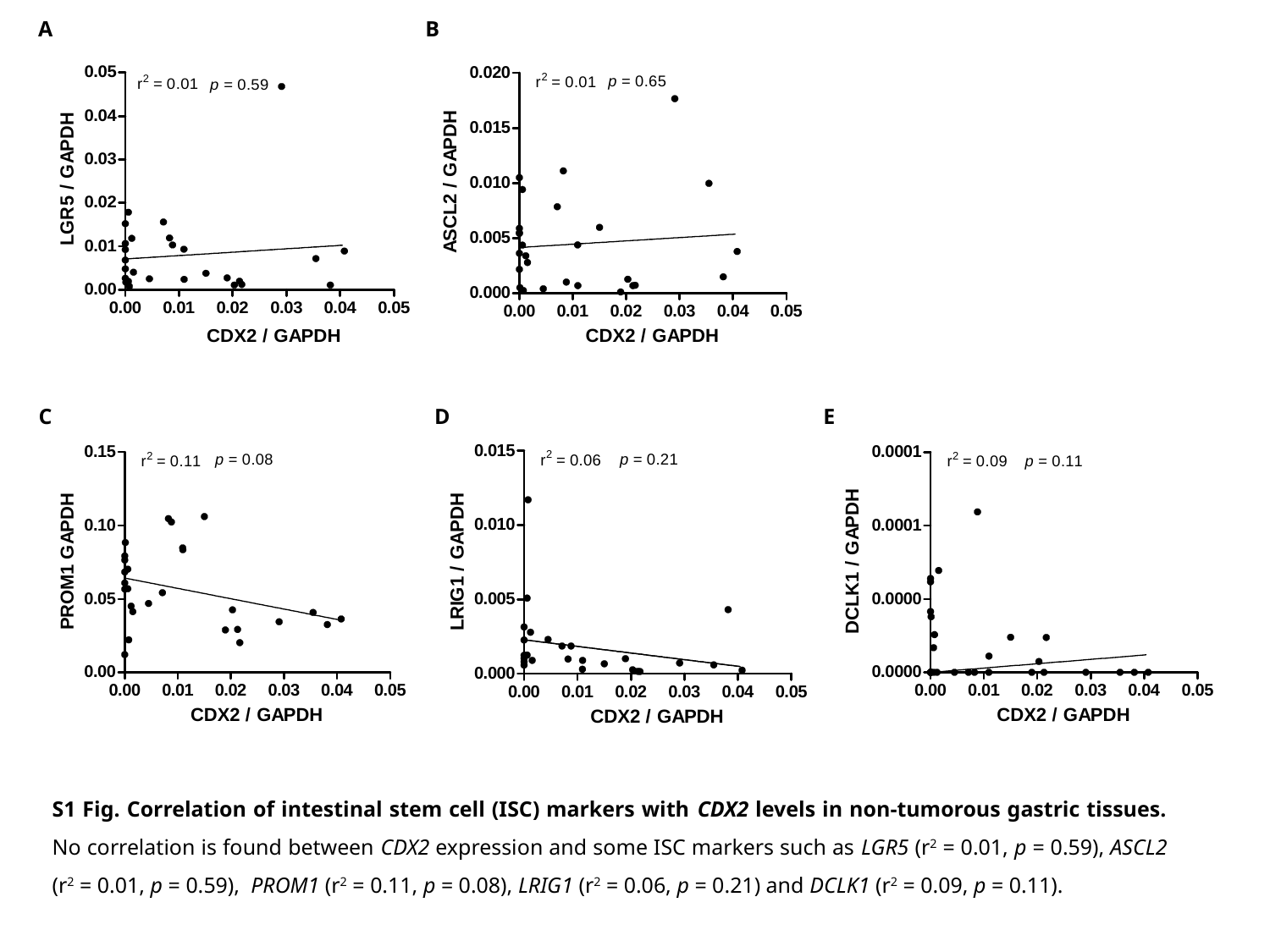

A
B
C
D
E
S1 Fig. Correlation of intestinal stem cell (ISC) markers with CDX2 levels in non-tumorous gastric tissues. No correlation is found between CDX2 expression and some ISC markers such as LGR5 (r2 = 0.01, p = 0.59), ASCL2 (r2 = 0.01, p = 0.59), PROM1 (r2 = 0.11, p = 0.08), LRIG1 (r2 = 0.06, p = 0.21) and DCLK1 (r2 = 0.09, p = 0.11).
