# Supplementary material for: Intestinal Stem Cell Markers in the Intestinal Metaplasia of Stomach and Barrett’s Esophagus
Source: PLoS One. 2015 May 21;10(5):e0127300. doi: 10.1371/journal.pone.0127300 (PMC4440782; doi:10.1371/journal.pone.0127300)
Supplement: S2 Fig — RNA ISH performed on a formalin-fixed and paraffin-embedded specimen of small intestine. (A, B) A group of LGR5 + stem cells are identified at the bottom of all crypts, intermingled with Paneth cells. Other intestinal stem cell markers such as ASCL2 (C, D), EPHB2 (E, F), and OLFM4 (G, H) are also found to be confined to the crypt bases. Magnification: A, C, E, G ×100; B, D, F, H ×400. (PPTX) [file pone.0127300.s002.pptx]

## Slide 1
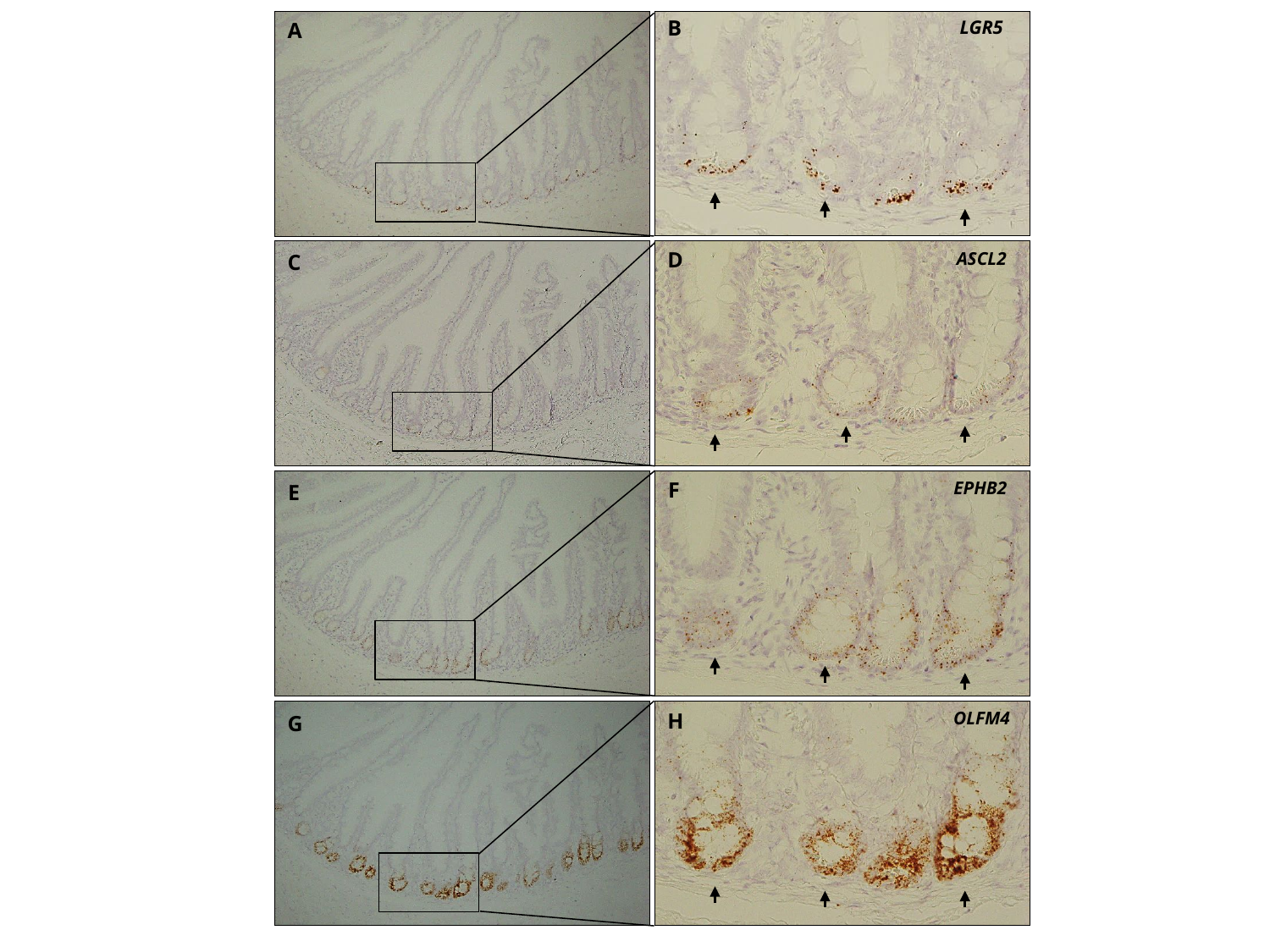

B
LGR5
A
D
ASCL2
C
F
EPHB2
E
OLFM4
H
G

## Slide 2
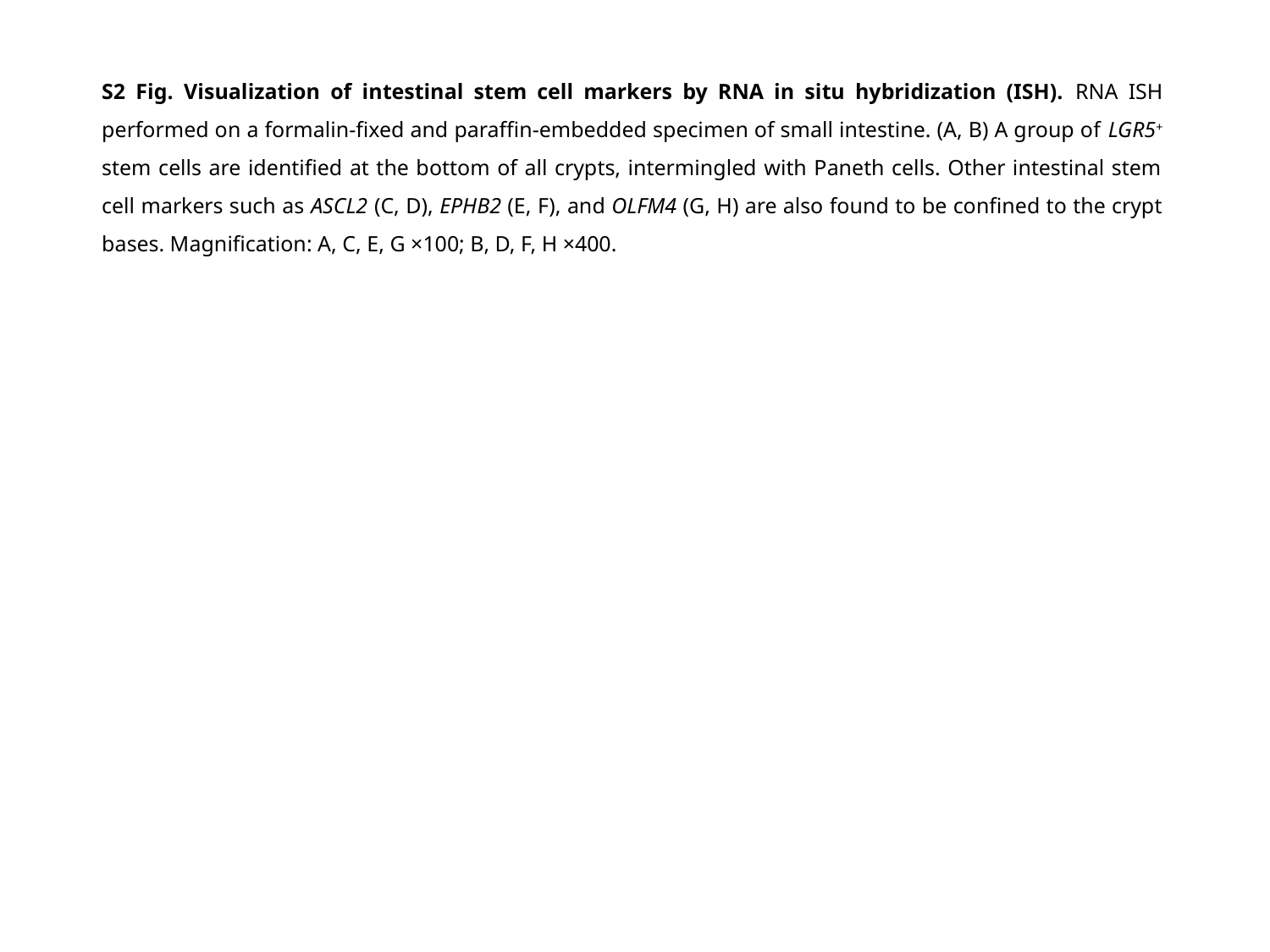

S2 Fig. Visualization of intestinal stem cell markers by RNA in situ hybridization (ISH). RNA ISH performed on a formalin-fixed and paraffin-embedded specimen of small intestine. (A, B) A group of LGR5+ stem cells are identified at the bottom of all crypts, intermingled with Paneth cells. Other intestinal stem cell markers such as ASCL2 (C, D), EPHB2 (E, F), and OLFM4 (G, H) are also found to be confined to the crypt bases. Magnification: A, C, E, G ×100; B, D, F, H ×400.
