# Supplementary material for: Intestinal Stem Cell Markers in the Intestinal Metaplasia of Stomach and Barrett’s Esophagus
Source: PLoS One. 2015 May 21;10(5):e0127300. doi: 10.1371/journal.pone.0127300 (PMC4440782; doi:10.1371/journal.pone.0127300)
Supplement: S3 Fig — Remaining gastric glands are frequently found at the basal areas of GI type IM (A and B). RNA ISH shows that LGR5 (C) and EPHB2 (D) expressions are localized above the gastric glands. Interestingly, OLFM4 (E) expression is observed in the gastric glands as well although its intensity is much weaker than that in the metaplastic glands. When those gastric glands disappear as IM develops (A and F), the distribution of all LGR5 (G), EPHB2 (H) and OLFM4 (I) is strictly confined to the basal areas. Arrows indicate the remaining gastric glands. Magnification: A ×40; B, C, D, E, F, G, H, I ×200. (PPTX) [file pone.0127300.s003.pptx]

## Slide 1
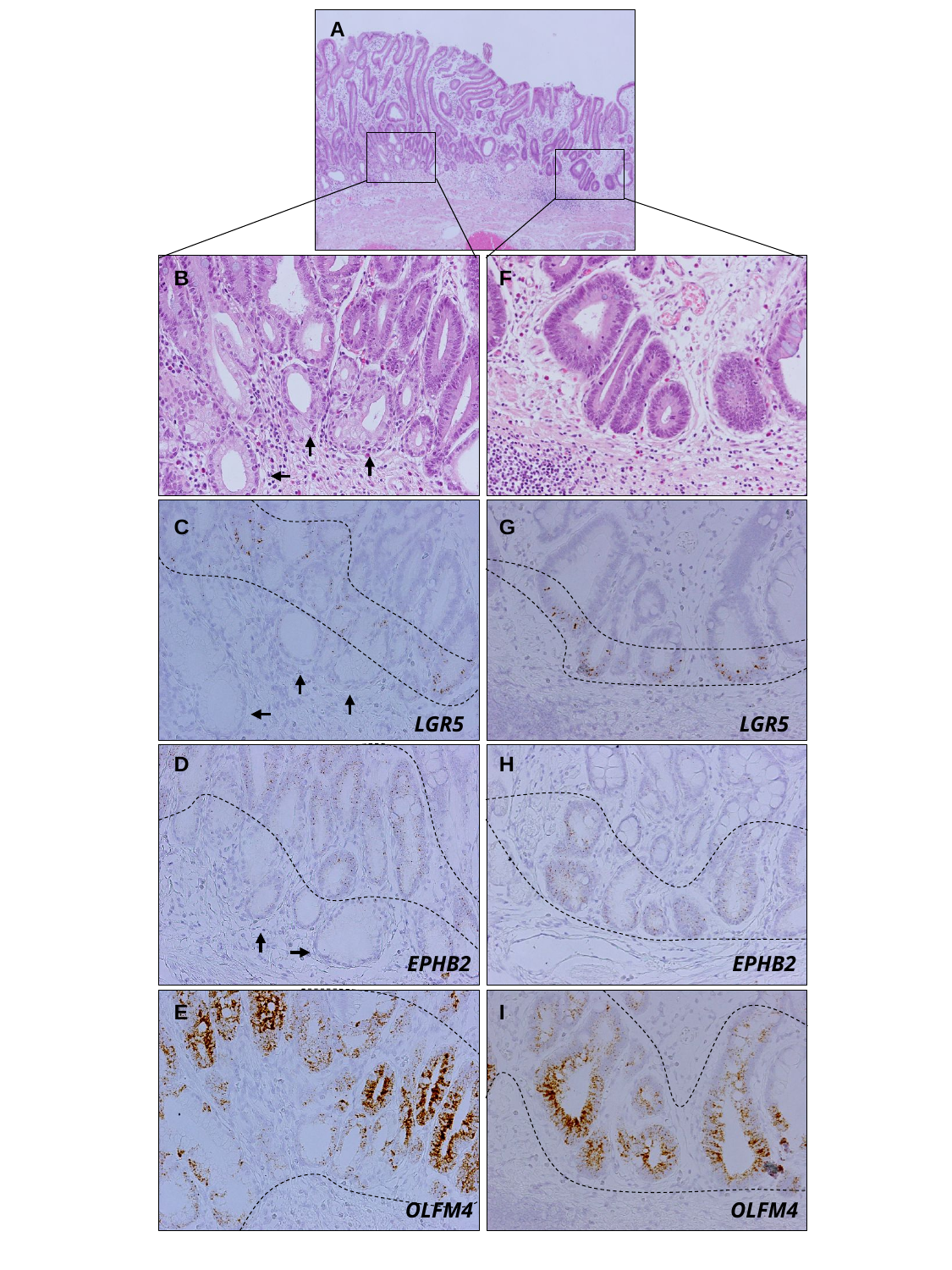

A
B
F
C
G
LGR5
LGR5
D
H
EPHB2
EPHB2
E
I
OLFM4
OLFM4

## Slide 2
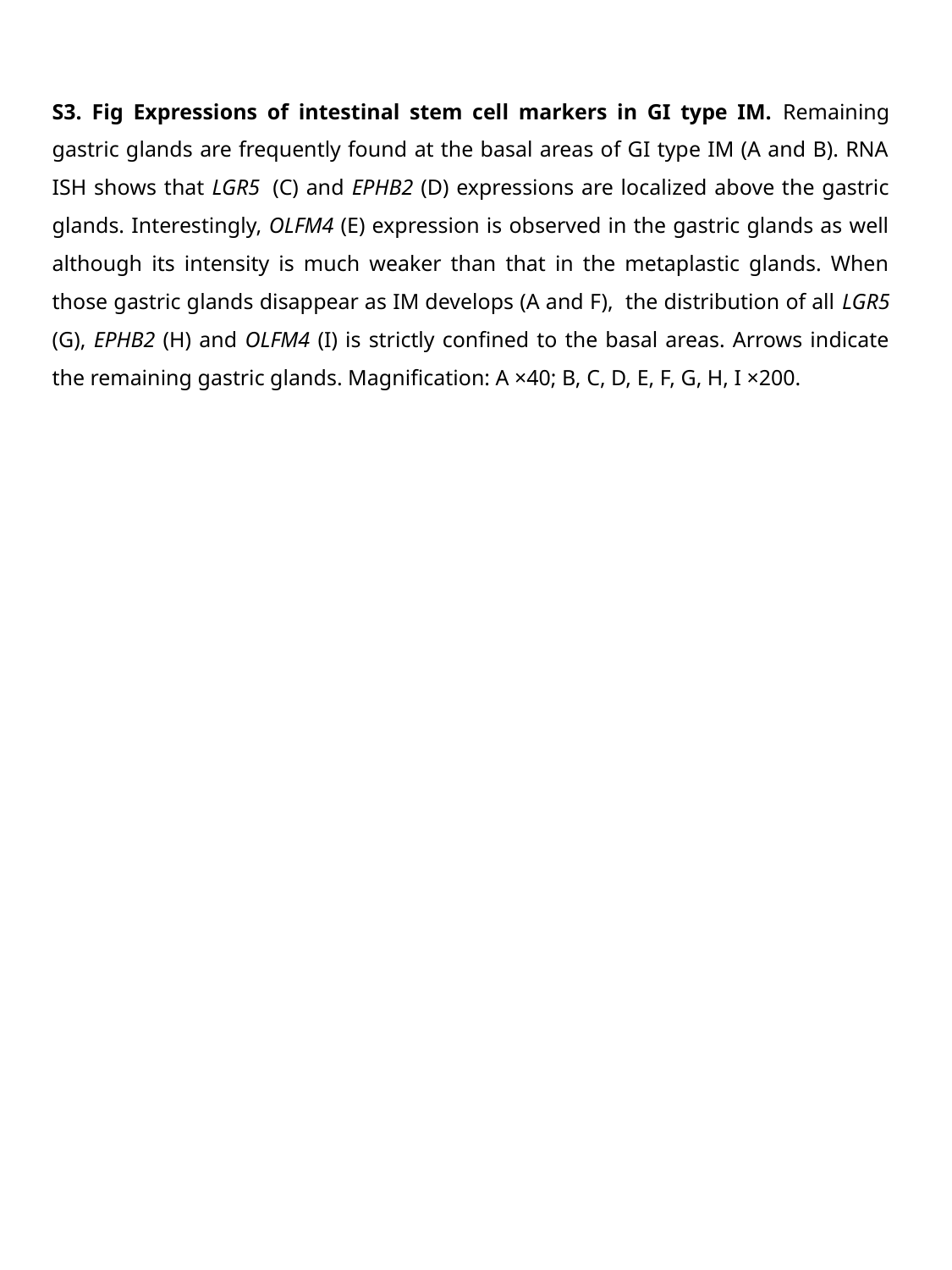

S3. Fig Expressions of intestinal stem cell markers in GI type IM. Remaining gastric glands are frequently found at the basal areas of GI type IM (A and B). RNA ISH shows that LGR5 (C) and EPHB2 (D) expressions are localized above the gastric glands. Interestingly, OLFM4 (E) expression is observed in the gastric glands as well although its intensity is much weaker than that in the metaplastic glands. When those gastric glands disappear as IM develops (A and F), the distribution of all LGR5 (G), EPHB2 (H) and OLFM4 (I) is strictly confined to the basal areas. Arrows indicate the remaining gastric glands. Magnification: A ×40; B, C, D, E, F, G, H, I ×200.
