# Supplementary material for: Intestinal Stem Cell Markers in the Intestinal Metaplasia of Stomach and Barrett’s Esophagus
Source: PLoS One. 2015 May 21;10(5):e0127300. doi: 10.1371/journal.pone.0127300 (PMC4440782; doi:10.1371/journal.pone.0127300)
Supplement: S4 Fig — (A and B) A small focus of IM in the middle of fundic glands, indicated by arrows, shows the same expression patterns of LGR5 (C), ASCL2 (D), and OLFM4 (E) as the IM of antrum. Magnifications: A ×100; B, C, D, E ×200. (PPTX) [file pone.0127300.s004.pptx]

## Slide 1
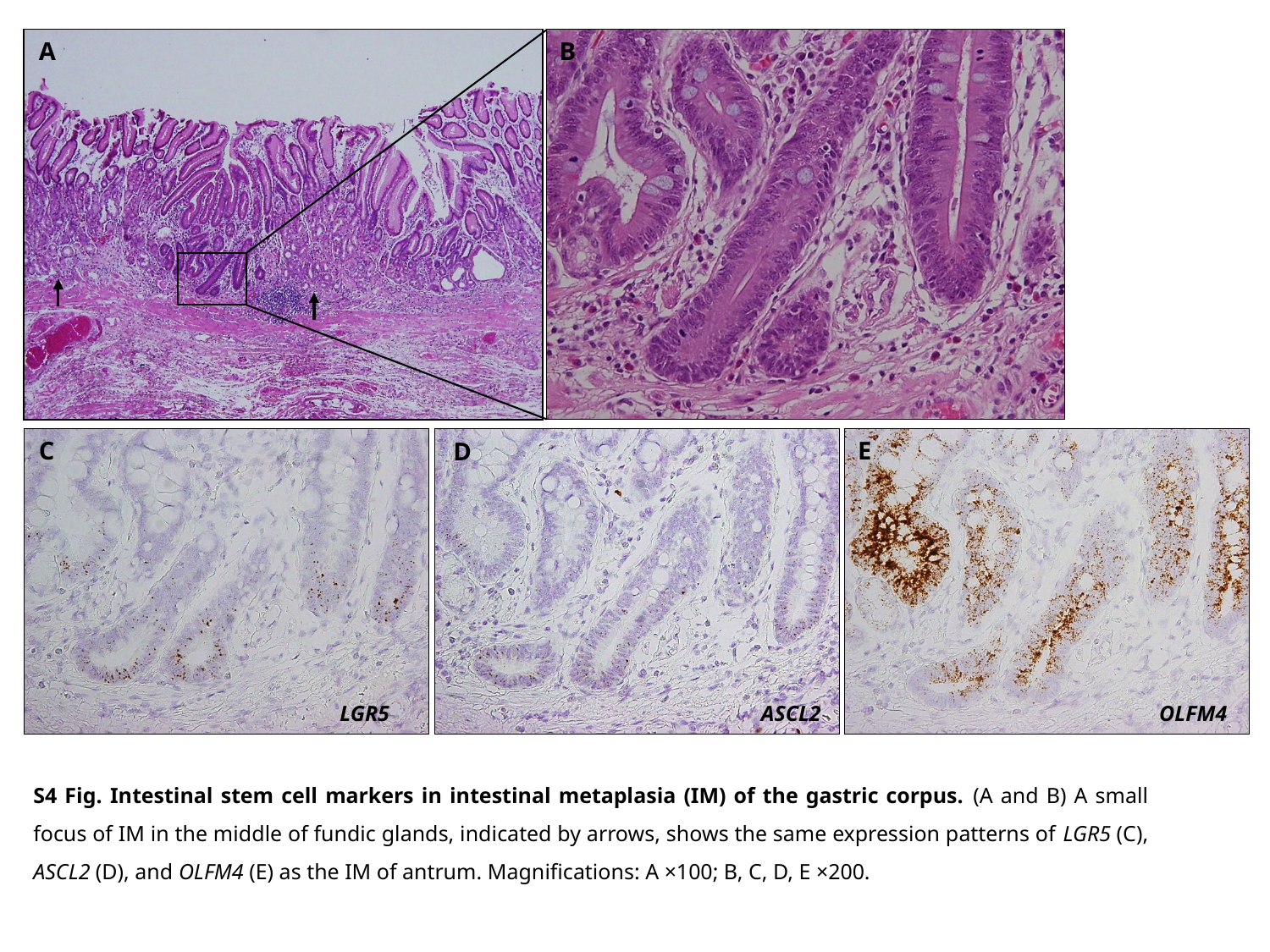

B
A
E
C
D
LGR5
ASCL2
OLFM4
S4 Fig. Intestinal stem cell markers in intestinal metaplasia (IM) of the gastric corpus. (A and B) A small focus of IM in the middle of fundic glands, indicated by arrows, shows the same expression patterns of LGR5 (C), ASCL2 (D), and OLFM4 (E) as the IM of antrum. Magnifications: A ×100; B, C, D, E ×200.
