# Supplementary material for: Intestinal Stem Cell Markers in the Intestinal Metaplasia of Stomach and Barrett’s Esophagus
Source: PLoS One. 2015 May 21;10(5):e0127300. doi: 10.1371/journal.pone.0127300 (PMC4440782; doi:10.1371/journal.pone.0127300)
Supplement: S5 Fig — Transfection of CDX2 into four GC cell lines, MKN74 (A), MKN28 (B), SNU484 (C) and SNU668 (D) significantly increases the amount of mRNA of CDX2 (**, p < 0.01; ***, p < 0.005). The EPHB2 expression is only marginally enhanced by the expression of CDX2 in three of four GC cell lines (***, p < 0.005). No difference is found in the levels of LGR5, ASCL2, and OLFM4 upon CDX2 overexpression (ns, not significant). (PPTX) [file pone.0127300.s005.pptx]

## Slide 1
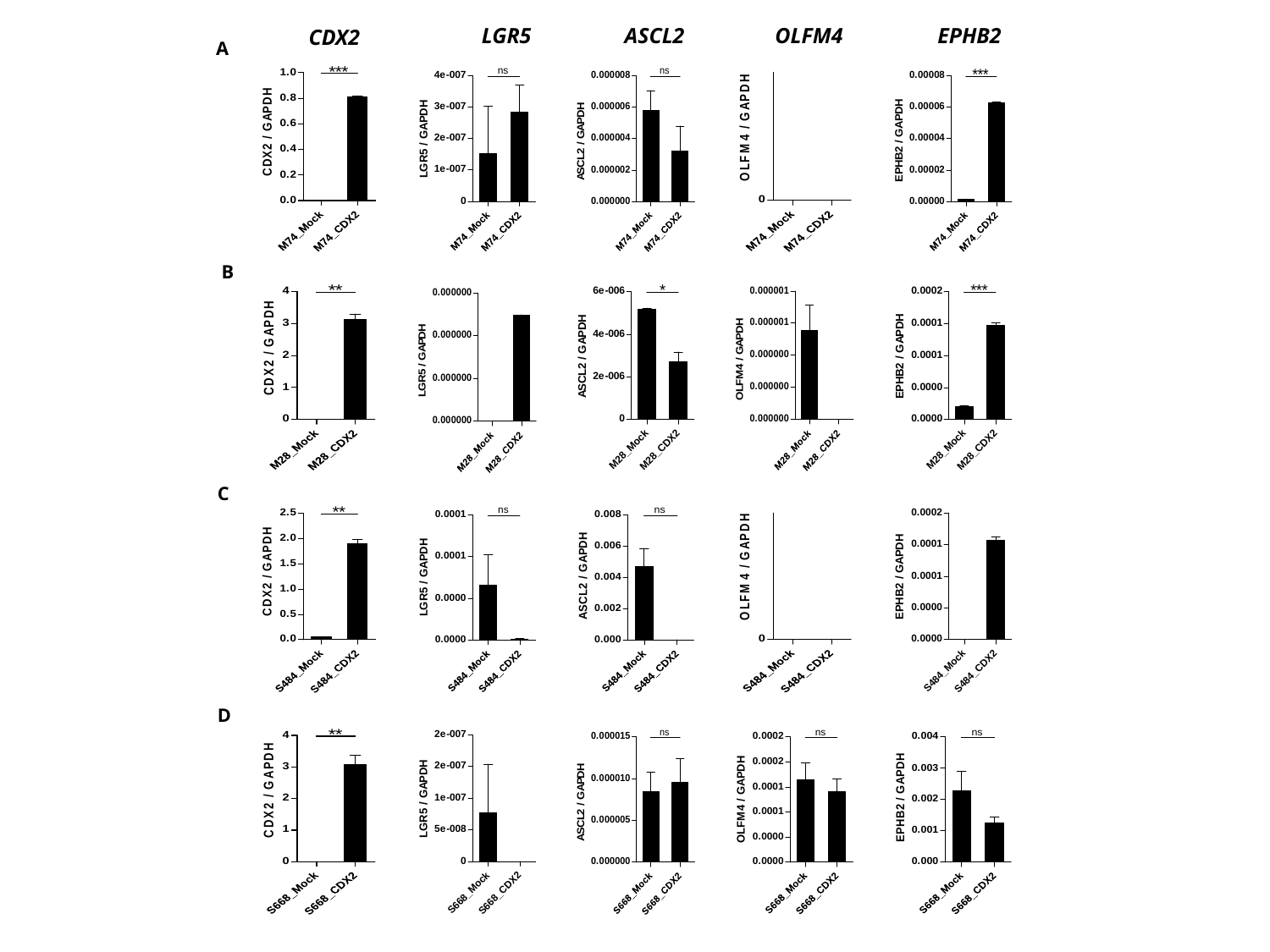

ASCL2
EPHB2
LGR5
OLFM4
CDX2
A
B
C
D

## Slide 2
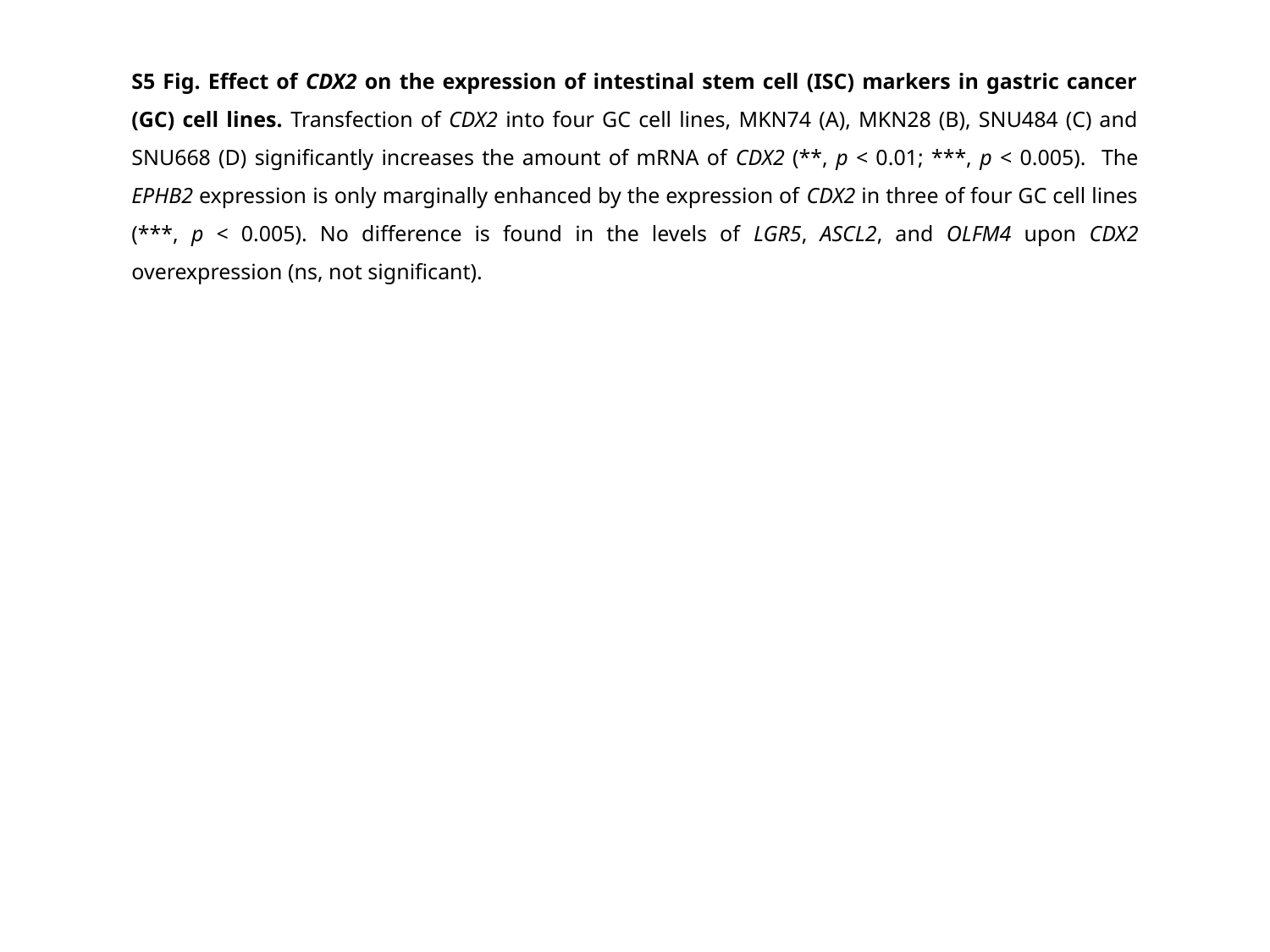

S5 Fig. Effect of CDX2 on the expression of intestinal stem cell (ISC) markers in gastric cancer (GC) cell lines. Transfection of CDX2 into four GC cell lines, MKN74 (A), MKN28 (B), SNU484 (C) and SNU668 (D) significantly increases the amount of mRNA of CDX2 (**, p < 0.01; ***, p < 0.005). The EPHB2 expression is only marginally enhanced by the expression of CDX2 in three of four GC cell lines (***, p < 0.005). No difference is found in the levels of LGR5, ASCL2, and OLFM4 upon CDX2 overexpression (ns, not significant).
